# Crystal structure of (2-formyl­phenolato-κ^2^ *O*,*O*′)oxido(2-{[(2-oxidoeth­yl)imino]­meth­yl}phenolato-κ^3^ *O*,*N*,*O*′)vanadium(V)

**DOI:** 10.1107/S2056989015006477

**Published:** 2015-04-09

**Authors:** Sowmianarayanan Parimala, Parasuraman Selvam

**Affiliations:** a National Centre for Catalysis Research, Chemistry Department, Indian Institute of Technology-Madras, Chennai 600 032, India; b New Industry Creation Hatchery Center, Tohoku University, Sendai 980 8579, Japan; c School of Science and Health, University of Western, Sydney, Penrith, NSW 275, Australia

**Keywords:** crystal structure, vanadyl complex, catalyst, hydrogen bonding, C—H⋯π inter­actions

## Abstract

In the unsymmetrical title vanadyl complex, [V(C_9_H_9_NO_2_)(C_7_H_5_O_2_)O], one of the ligands (2-formyl­phenol) is disordered over two sets of sites, with an occupancy ratio of 0.55 (2):0.45 (2). The metal atom is hexa­coordinated, with a distorted octa­hedral geometry. The vanadyl O atom (which subtends the shortest V—O bond) occupies one of the apical positions and the remaining axial bond (the longest in the polyhedron) is provided by the (disordered) formyl O atoms. The basal plane is defined by the two phenoxide O atoms, the imino­alcoholic O and the imino N atom. The planes of the two benzene rings are almost perpendicular to each other, subtending an inter­planar angle of 84.1 (2)° between the major parts. The crystal structure features weak C—H⋯O and C—H⋯π inter­actions, forming a lateral arrangement of adjacent molecules.

## Related literature   

For general background to catalysis, see: Forzatti *et al.* (1987 ▸); Harding *et al.* (1994 ▸); Xia *et al.* (2012 ▸); Salavati-Niasari *et al.* (2004 ▸). For C—H oxidation reactions, see: Grivani *et al.* (2013 ▸); Maurya *et al.* (2011 ▸); Talsi *et al.* (1993 ▸); Zhang *et al.* (2005 ▸). 
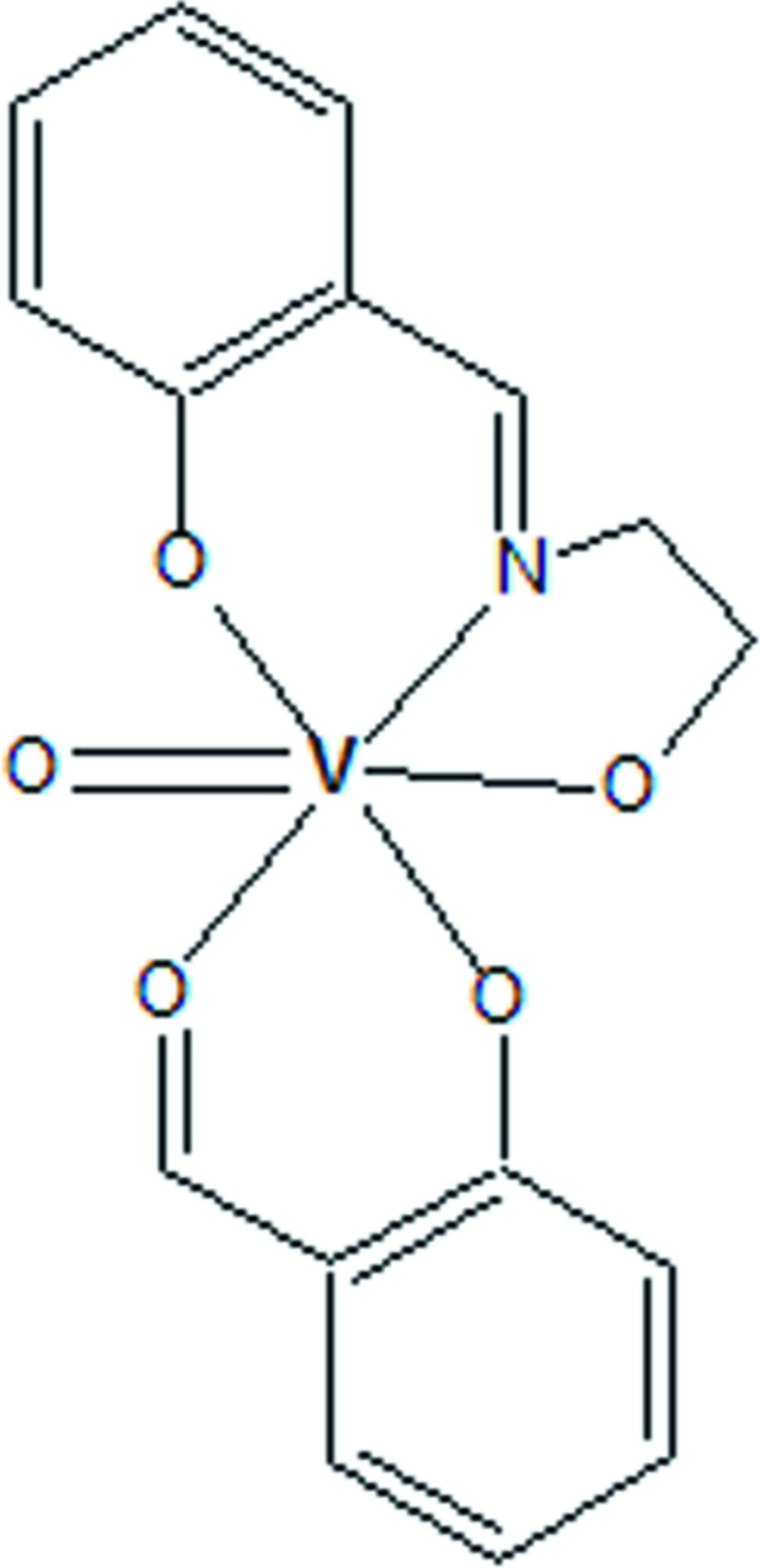


## Experimental   

### Crystal data  

[V(C_9_H_9_NO_2_)(C_7_H_5_O_2_)O]
*M* *_r_* = 351.22
Monoclinic, 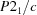

*a* = 6.6915 (2) Å
*b* = 7.6542 (4) Å
*c* = 29.1847 (9) Å
β = 95.126 (3)°
*V* = 1488.81 (10) Å^3^
*Z* = 4
Mo *K*α radiation
μ = 0.69 mm^−1^
*T* = 293 K
0.25 × 0.25 × 0.20 mm

### Data collection  

Bruker Kappa APEXII CCD diffractometer
Absorption correction: multi-scan (*SADABS*; Bruker, 2004 ▸) *T*_min_ = 0.846, *T*_max_ = 0.880
18824 measured reflections
3249 independent reflections
2918 reflections with *I* > 2σ(*I*)
*R*_int_ = 0.025

### Refinement  

*R*[*F* ^2^ > 2σ(*F* ^2^)] = 0.044
*wR*(*F* ^2^) = 0.102
*S* = 1.22
3249 reflections
290 parameters
143 restraints
H-atom parameters constrained
Δρ_max_ = 0.29 e Å^−3^
Δρ_min_ = −0.35 e Å^−3^

### 

Data collection: *APEX2* (Bruker, 2004 ▸); cell refinement: *APEX2* and *SAINT* (Bruker, 2004 ▸); data reduction: *SAINT* and *XPREP* (Bruker, 2004 ▸); program(s) used to solve structure: *SIR92* (Altomare *et al.*, 1993 ▸); program(s) used to refine structure: *SHELXL2014/7* (Sheldrick, 2015 ▸); molecular graphics: *ORTEP-3 for Windows* (Farrugia, 2012 ▸) and *Mercury* (Macrae *et al.*, 2006 ▸); software used to prepare material for publication: *SHELXL2014/7*.

## Supplementary Material

Crystal structure: contains datablock(s) I, New_Global_Publ_Block. DOI: 10.1107/S2056989015006477/bg2547sup1.cif

Structure factors: contains datablock(s) I. DOI: 10.1107/S2056989015006477/bg2547Isup2.hkl

**Fig. 1.**
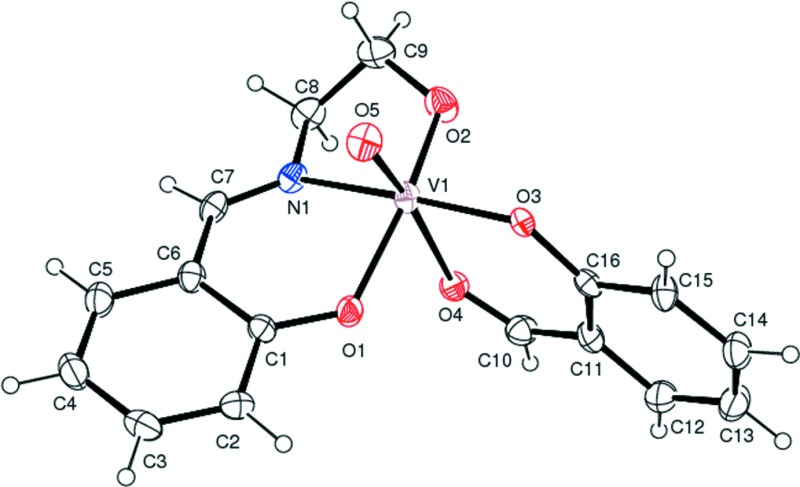
Molecular structure of the title compound with atom labelling. Displacement ellipsoids are drawn at 30% probability level. (The minor component of the disordered 2-formylphenol moiety is not shown).

. DOI: 10.1107/S2056989015006477/bg2547fig1.tif
Mol­ecular structure of the title compound with atom labelling. Displacement ellipsoids are drawn at 30% probability level. (The minor component of the disordered 2-formyl­phenol moiety is not shown).

CCDC reference: 1057002

Additional supporting information:  crystallographic information; 3D view; checkCIF report

## Figures and Tables

**Table 1:** Selected bond lengths ()

V1O5	1.5886(19)
V1O3	1.859(3)
V1O2	1.8333(18)
V1O1	1.8957(17)
V1O3	1.859(3)
V1N1	2.0966(19)
V1O4	2.269(4)
V1O4	2.275(3)

**Table 2:** Hydrogen-bond geometry (, ) *Cg* is the centroid of the C1C6 ring.

*D*H*A*	*D*H	H*A*	*D* *A*	*D*H*A*
C3H3O5^i^	0.93	2.56	3.352(4)	143
C5H5O4^ii^	0.93	2.50	3.220(13)	134
C2H2*Cg* ^i^	0.93	2.84	3.615(3)	141

Symmetry codes: (i) 
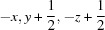
; (ii) 
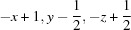
.

## supplementary crystallographic information

### S1. Introduction

Vanadyl complexes are good catalysts for oxidation of organic compounds as they have the ability to transfer an oxygen atom for epoxidation and C—H oxidation reactions by hydrogen peroxide and tertiary butyl hydro­peroxide. In the presence of peroxides they are readily converted into oxoperoxovanadium(V) complexes or alkyl­hydro­peroxideVanadium(V) complex.

### S2.1. Synthesis and crystallization

The title complex was synthesized by the reaction of Vanadylsulphate ( 4.52g, 0.025mol) in 20ml of methanol with 2-formyl­phenol ( 5.24ml, 0.05mol) in 20ml of methanol and the mixture was refluxed for 1 hr. To the above mixture, 10ml of methanol containing 2-amino­ethanol (1.5ml, 0.025mol) was added and the reflux was continued for 6 hr. The resulting dark brown crystalline solid was filtered, washed quickly with cold methanol and recrystallized from methanol to get pure blackish brown crystals.

### S2.2. Refinement

All the hydrogen atoms in the molecule were identified from the difference electron density map, further idealized and treated as riding with a distance d(C—H)=0.93Å (for aromatic C—H) and d(C—H)=0.97 for (CH_2_)respectively. In all cases Uiso(H)=-1.2Ueq.

The 2-formyl­phenyl moeity is disordered over two sites, with site occupancies 0.55:0.452 (2). C—C and C—O bond distances in the disordered moieties were restrained using similarity restraints (SAME command in SHELXL2014), while continuity restraints were applied to the anisotropic displacement parameters Uij for all atoms (RIGU command in SHELXL2014).

### S3. Results and discussion

Crystal data, data collection and structure refinement details for the title compound C_16_H_14_NO_5_V (I) are summarized in Table 1, while bond distances are summarized in Table 2. An ORTEP diagram of the molecule with 30% probability displacement factors is shown in Figure 1.

The molecule consists of a vanadyl moiety with (O,O') 2-formyl­phenol and (O,N,O') ((2-hy­droxy­ethyl­imino)­methyl)­phenol ligands bound to vanadium and defining a distorted o­cta­hedral geometry around the cation. The (2-formyl­phenol) group is disordered over two sites with occupancies of 55:45 (2). The basal plane of the (distorted) vanadium o­cta­hedral environment is defined by phenoxide oxygens O1 (C1—C6) and O3 (C11—C16), the imino N1 and the imino­alcoholic O2. The vanadyl oxygen O5 (which subtends the shortest V—O bond, see Table 2) occupies one of the apical positions; this axial oxygen atom is suitable for the formation of a cyclic V—O—O inter­mediate which is an important step in the mechanism of peroxidative oxidation of organic compounds. The remaining axial bond is provided by O4 from the formyl group belonging to the phenyl ring C11—C16, and which displays the longest V—O distance in the molecule. The planes of the two phenyl rings are almost perpendicular to each other, subtending an inter­planar angle of 84.1 (2)^ο^^.^ between major parts.

The crystal structure is stabilized by some weak C—H···O and C—H···π contacts presented in Table 3.

### Crystal data

**Table:**

[V(C_9_H_9_NO_2_)(C_7_H_5_O_2_)O]	*F*(000) = 720
*M**_r_* = 351.22	*D*_x_ = 1.567 Mg m^−^^3^
Monoclinic, *P*2_1_/*c*	Mo *K*α radiation, λ = 0.71073 Å
*a* = 6.6915 (2) Å	Cell parameters from 7573 reflections
*b* = 7.6542 (4) Å	θ = 2.8–29.9°
*c* = 29.1847 (9) Å	µ = 0.69 mm^−^^1^
β = 95.126 (3)°	*T* = 293 K
*V* = 1488.81 (10) Å^3^	Blocks, black
*Z* = 4	0.25 × 0.25 × 0.20 mm

### Data collection

**Table:**

Bruker Kappa APEXII CCD diffractometer	2918 reflections with *I* > 2σ(*I*)
Radiation source: fine-focus sealed tube	*R*_int_ = 0.025
ω and φ scan	θ_max_ = 27.0°, θ_min_ = 2.8°
Absorption correction: multi-scan (*SADABS*; Bruker, 2004)	*h* = −8→8
*T*_min_ = 0.846, *T*_max_ = 0.880	*k* = −9→9
18824 measured reflections	*l* = −37→37
3249 independent reflections	

### Refinement

**Table:**

Refinement on *F*^2^	143 restraints
Least-squares matrix: full	Hydrogen site location: inferred from neighbouring sites
*R*[*F*^2^ > 2σ(*F*^2^)] = 0.044	H-atom parameters constrained
*wR*(*F*^2^) = 0.102	*w* = 1/[σ^2^(*F*_o_^2^) + (0.0298*P*)^2^ + 1.478*P*] where *P* = (*F*_o_^2^ + 2*F*_c_^2^)/3
*S* = 1.22	(Δ/σ)_max_ = 0.001
3249 reflections	Δρ_max_ = 0.29 e Å^−^^3^
290 parameters	Δρ_min_ = −0.35 e Å^−^^3^

### Special details

**Table:**

Geometry. All e.s.d.'s (except the e.s.d. in the dihedral angle between two l.s. planes) are estimated using the full covariance matrix. The cell e.s.d.'s are taken into account individually in the estimation of e.s.d.'s in distances, angles and torsion angles; correlations between e.s.d.'s in cell parameters are only used when they are defined by crystal symmetry. An approximate (isotropic) treatment of cell e.s.d.'s is used for estimating e.s.d.'s involving l.s. planes.

### Fractional atomic coordinates and isotropic or equivalent isotropic displacement parameters (Å^2^)

**Table:**

	*x*	*y*	*z*	*U*_iso_*/*U*_eq_	Occ. (<1)
V1	0.26635 (5)	0.25635 (6)	0.12647 (2)	0.02941 (13)	
N1	0.5270 (3)	0.2221 (3)	0.17101 (7)	0.0309 (4)	
O1	0.1761 (2)	0.3678 (2)	0.17886 (5)	0.0335 (4)	
O2	0.4526 (3)	0.2057 (3)	0.08602 (6)	0.0441 (5)	
O5	0.1870 (3)	0.0660 (3)	0.13678 (7)	0.0430 (4)	
C1	0.1947 (3)	0.3159 (3)	0.22234 (8)	0.0283 (5)	
C2	0.0450 (4)	0.3559 (3)	0.25122 (9)	0.0353 (5)	
H2	−0.0677	0.4184	0.2398	0.042*	
C3	0.0638 (4)	0.3033 (4)	0.29651 (9)	0.0430 (6)	
H3	−0.0391	0.3274	0.3150	0.052*	
C4	0.2328 (5)	0.2150 (4)	0.31514 (9)	0.0450 (7)	
H4	0.2430	0.1793	0.3457	0.054*	
C5	0.3843 (4)	0.1810 (4)	0.28796 (9)	0.0392 (6)	
H5	0.5002	0.1259	0.3006	0.047*	
C6	0.3684 (3)	0.2278 (3)	0.24135 (8)	0.0315 (5)	
C7	0.5346 (3)	0.1987 (3)	0.21439 (9)	0.0334 (5)	
H7	0.6550	0.1606	0.2295	0.040*	
C8	0.7059 (4)	0.1994 (4)	0.14621 (10)	0.0395 (6)	
H8A	0.8031	0.1246	0.1633	0.047*	
H8B	0.7682	0.3111	0.1409	0.047*	
C9	0.6286 (4)	0.1157 (4)	0.10163 (10)	0.0438 (6)	
H9A	0.7273	0.1248	0.0793	0.053*	
H9B	0.5998	−0.0069	0.1063	0.053*	
O3	0.0652 (12)	0.3279 (8)	0.0824 (4)	0.0280 (19)	0.55 (2)
O4	0.3721 (14)	0.5368 (8)	0.1196 (5)	0.035 (2)	0.55 (2)
C10	0.2794 (14)	0.6543 (7)	0.0990 (4)	0.0368 (19)	0.55 (2)
H10	0.3396	0.7639	0.1000	0.044*	0.55 (2)
C11	0.0863 (13)	0.6404 (7)	0.0732 (3)	0.0310 (16)	0.55 (2)
C12	0.0013 (15)	0.7914 (8)	0.0525 (3)	0.043 (2)	0.55 (2)
H12	0.0660	0.8983	0.0572	0.052*	0.55 (2)
C13	−0.1765 (14)	0.7829 (11)	0.0252 (3)	0.044 (2)	0.55 (2)
H13	−0.2320	0.8833	0.0114	0.053*	0.55 (2)
C14	−0.2714 (11)	0.6253 (12)	0.0186 (3)	0.0386 (18)	0.55 (2)
H14	−0.3922	0.6200	0.0002	0.046*	0.55 (2)
C15	−0.1924 (9)	0.4741 (11)	0.0386 (3)	0.0338 (17)	0.55 (2)
H15	−0.2611	0.3690	0.0340	0.041*	0.55 (2)
C16	−0.0087 (11)	0.4785 (8)	0.0657 (4)	0.0262 (16)	0.55 (2)
O3'	0.0547 (14)	0.3519 (9)	0.0891 (5)	0.028 (2)	0.45 (2)
O4'	0.4058 (16)	0.5227 (10)	0.1170 (6)	0.039 (3)	0.45 (2)
C10'	0.3244 (16)	0.6488 (8)	0.0978 (5)	0.041 (3)	0.45 (2)
H10'	0.3946	0.7538	0.1001	0.050*	0.45 (2)
C11'	0.1309 (15)	0.6516 (8)	0.0720 (5)	0.037 (2)	0.45 (2)
C12'	0.0638 (18)	0.8090 (8)	0.0514 (4)	0.043 (2)	0.45 (2)
H12'	0.1444	0.9080	0.0547	0.052*	0.45 (2)
C13'	−0.1199 (18)	0.8183 (11)	0.0266 (4)	0.045 (2)	0.45 (2)
H13'	−0.1619	0.9218	0.0121	0.054*	0.45 (2)
C14'	−0.2409 (14)	0.6738 (14)	0.0234 (4)	0.043 (2)	0.45 (2)
H14'	−0.3661	0.6809	0.0069	0.052*	0.45 (2)
C15'	−0.1817 (13)	0.5178 (13)	0.0441 (4)	0.039 (2)	0.45 (2)
H15'	−0.2659	0.4211	0.0410	0.047*	0.45 (2)
C16'	0.0050 (13)	0.5050 (10)	0.0697 (5)	0.031 (2)	0.45 (2)

### Atomic displacement parameters (Å^2^)

**Table:**

	*U*^11^	*U*^22^	*U*^33^	*U*^12^	*U*^13^	*U*^23^
V1	0.0230 (2)	0.0370 (2)	0.0279 (2)	−0.00340 (17)	0.00052 (14)	0.00299 (17)
N1	0.0210 (9)	0.0339 (11)	0.0381 (11)	0.0003 (8)	0.0031 (8)	0.0045 (9)
O1	0.0282 (8)	0.0432 (10)	0.0289 (8)	0.0098 (7)	0.0016 (6)	0.0045 (7)
O2	0.0359 (9)	0.0629 (13)	0.0347 (9)	0.0014 (9)	0.0096 (8)	−0.0020 (9)
O5	0.0384 (10)	0.0422 (11)	0.0479 (11)	−0.0069 (8)	0.0020 (8)	0.0045 (9)
C1	0.0277 (11)	0.0292 (11)	0.0275 (11)	−0.0026 (9)	−0.0002 (9)	−0.0030 (9)
C2	0.0324 (13)	0.0342 (13)	0.0398 (13)	0.0035 (10)	0.0056 (10)	−0.0041 (11)
C3	0.0520 (16)	0.0416 (15)	0.0376 (14)	−0.0031 (13)	0.0153 (12)	−0.0097 (12)
C4	0.0621 (18)	0.0452 (16)	0.0273 (12)	−0.0051 (14)	0.0025 (12)	−0.0001 (11)
C5	0.0418 (14)	0.0405 (14)	0.0336 (13)	0.0004 (12)	−0.0058 (11)	0.0037 (11)
C6	0.0299 (11)	0.0329 (13)	0.0306 (12)	−0.0031 (10)	−0.0024 (9)	0.0016 (10)
C7	0.0226 (11)	0.0355 (13)	0.0407 (13)	0.0017 (9)	−0.0057 (9)	0.0059 (11)
C8	0.0230 (11)	0.0460 (15)	0.0508 (16)	0.0018 (11)	0.0102 (10)	0.0048 (12)
C9	0.0386 (14)	0.0441 (15)	0.0513 (16)	0.0002 (12)	0.0186 (12)	−0.0007 (13)
O3	0.030 (3)	0.036 (2)	0.017 (3)	−0.0034 (18)	−0.002 (2)	−0.002 (2)
O4	0.026 (3)	0.040 (3)	0.036 (3)	−0.007 (2)	−0.003 (3)	−0.002 (2)
C10	0.034 (3)	0.038 (3)	0.037 (3)	−0.0057 (17)	−0.005 (3)	−0.004 (2)
C11	0.031 (3)	0.037 (2)	0.024 (3)	−0.0048 (15)	0.000 (2)	−0.0031 (16)
C12	0.042 (4)	0.036 (3)	0.048 (3)	−0.007 (2)	−0.013 (3)	0.000 (2)
C13	0.041 (4)	0.038 (3)	0.048 (3)	−0.004 (2)	−0.014 (3)	0.000 (2)
C14	0.040 (3)	0.040 (3)	0.033 (3)	−0.005 (2)	−0.012 (2)	0.000 (2)
C15	0.032 (2)	0.039 (3)	0.028 (3)	−0.0076 (19)	−0.0067 (19)	0.001 (3)
C16	0.028 (2)	0.035 (2)	0.015 (3)	−0.0042 (15)	0.0015 (18)	−0.0029 (19)
O3'	0.026 (3)	0.035 (3)	0.022 (4)	−0.005 (2)	0.001 (2)	−0.002 (3)
O4'	0.029 (4)	0.048 (4)	0.039 (4)	−0.009 (3)	0.005 (3)	0.003 (3)
C10'	0.037 (4)	0.045 (4)	0.041 (4)	−0.011 (2)	−0.001 (3)	0.000 (3)
C11'	0.038 (3)	0.039 (3)	0.034 (4)	−0.008 (2)	0.003 (3)	−0.002 (2)
C12'	0.048 (4)	0.038 (3)	0.042 (4)	−0.009 (2)	−0.005 (4)	−0.001 (3)
C13'	0.049 (4)	0.039 (3)	0.046 (4)	−0.008 (3)	−0.007 (3)	−0.004 (3)
C14'	0.040 (4)	0.039 (3)	0.048 (5)	−0.004 (3)	−0.013 (3)	−0.001 (3)
C15'	0.040 (3)	0.038 (3)	0.037 (4)	−0.003 (2)	−0.008 (3)	−0.004 (3)
C16'	0.034 (3)	0.037 (3)	0.020 (4)	−0.0031 (19)	0.001 (2)	−0.001 (3)

### Geometric parameters (Å, º)

**Table:**

V1—O5	1.5886 (19)	O3—C16	1.330 (4)
V1—O3	1.859 (3)	O4—C10	1.220 (4)
V1—O2	1.8333 (18)	C10—C11	1.439 (5)
V1—O1	1.8957 (17)	C10—H10	0.9300
V1—O3'	1.859 (3)	C11—C12	1.402 (4)
V1—N1	2.0966 (19)	C11—C16	1.401 (5)
V1—O4'	2.269 (4)	C12—C13	1.372 (6)
V1—O4	2.275 (3)	C12—H12	0.9300
N1—C7	1.275 (3)	C13—C14	1.369 (7)
N1—C8	1.464 (3)	C13—H13	0.9300
O1—C1	1.325 (3)	C14—C15	1.379 (5)
O2—C9	1.404 (3)	C14—H14	0.9300
C1—C2	1.400 (3)	C15—C16	1.401 (4)
C1—C6	1.413 (3)	C15—H15	0.9300
C2—C3	1.377 (4)	O3'—C16'	1.330 (4)
C2—H2	0.9300	O4'—C10'	1.220 (4)
C3—C4	1.386 (4)	C10'—C11'	1.439 (5)
C3—H3	0.9300	C10'—H10'	0.9300
C4—C5	1.367 (4)	C11'—C12'	1.402 (4)
C4—H4	0.9300	C11'—C16'	1.401 (5)
C5—C6	1.401 (3)	C12'—C13'	1.372 (6)
C5—H5	0.9300	C12'—H12'	0.9300
C6—C7	1.436 (3)	C13'—C14'	1.369 (7)
C7—H7	0.9300	C13'—H13'	0.9300
C8—C9	1.500 (4)	C14'—C15'	1.380 (5)
C8—H8A	0.9700	C14'—H14'	0.9300
C8—H8B	0.9700	C15'—C16'	1.402 (4)
C9—H9A	0.9700	C15'—H15'	0.9300
C9—H9B	0.9700		
			
O5—V1—O3	99.65 (14)	C9—C8—H8B	110.9
O5—V1—O2	100.72 (10)	H8A—C8—H8B	108.9
O3—V1—O2	96.2 (5)	O2—C9—C8	106.6 (2)
O5—V1—O1	97.12 (9)	O2—C9—H9A	110.4
O3—V1—O1	99.3 (5)	C8—C9—H9A	110.4
O2—V1—O1	153.94 (8)	O2—C9—H9B	110.4
O5—V1—O3'	102.82 (18)	C8—C9—H9B	110.4
O2—V1—O3'	103.4 (6)	H9A—C9—H9B	108.6
O1—V1—O3'	90.9 (6)	C16—O3—V1	137.1 (4)
O5—V1—N1	92.39 (9)	C10—O4—V1	126.2 (3)
O3—V1—N1	167.5 (2)	O4—C10—C11	126.6 (3)
O2—V1—N1	78.28 (8)	O4—C10—H10	116.7
O1—V1—N1	82.15 (7)	C11—C10—H10	116.7
O3'—V1—N1	164.0 (2)	C12—C11—C16	120.1 (4)
O5—V1—O4'	174.29 (18)	C12—C11—C10	118.4 (4)
O2—V1—O4'	78.5 (5)	C16—C11—C10	121.4 (3)
O1—V1—O4'	81.8 (5)	C13—C12—C11	120.5 (4)
O3'—V1—O4'	82.83 (15)	C13—C12—H12	119.7
N1—V1—O4'	81.91 (16)	C11—C12—H12	119.7
O5—V1—O4	173.8 (4)	C12—C13—C14	119.4 (4)
O3—V1—O4	82.66 (13)	C12—C13—H13	120.3
O2—V1—O4	84.7 (4)	C14—C13—H13	120.3
O1—V1—O4	76.8 (4)	C13—C14—C15	121.6 (4)
N1—V1—O4	85.69 (13)	C13—C14—H14	119.2
C7—N1—C8	120.8 (2)	C15—C14—H14	119.2
C7—N1—V1	126.16 (16)	C14—C15—C16	120.1 (4)
C8—N1—V1	112.34 (16)	C14—C15—H15	119.9
C1—O1—V1	128.93 (15)	C16—C15—H15	119.9
C9—O2—V1	119.57 (16)	O3—C16—C15	117.7 (4)
O1—C1—C2	120.0 (2)	O3—C16—C11	124.1 (3)
O1—C1—C6	121.5 (2)	C15—C16—C11	118.2 (3)
C2—C1—C6	118.4 (2)	C16'—O3'—V1	138.1 (2)
C3—C2—C1	120.4 (2)	C10'—O4'—V1	126.5 (3)
C3—C2—H2	119.8	O4'—C10'—C11'	126.6 (3)
C1—C2—H2	119.8	O4'—C10'—H10'	116.7
C2—C3—C4	121.3 (3)	C11'—C10'—H10'	116.7
C2—C3—H3	119.3	C12'—C11'—C16'	120.0 (4)
C4—C3—H3	119.3	C12'—C11'—C10'	118.4 (4)
C5—C4—C3	119.1 (2)	C16'—C11'—C10'	121.4 (3)
C5—C4—H4	120.4	C13'—C12'—C11'	120.5 (4)
C3—C4—H4	120.4	C13'—C12'—H12'	119.7
C4—C5—C6	121.2 (2)	C11'—C12'—H12'	119.7
C4—C5—H5	119.4	C12'—C13'—C14'	119.4 (4)
C6—C5—H5	119.4	C12'—C13'—H13'	120.3
C5—C6—C1	119.5 (2)	C14'—C13'—H13'	120.3
C5—C6—C7	119.8 (2)	C13'—C14'—C15'	121.6 (4)
C1—C6—C7	120.6 (2)	C13'—C14'—H14'	119.2
N1—C7—C6	123.9 (2)	C15'—C14'—H14'	119.2
N1—C7—H7	118.0	C14'—C15'—C16'	120.1 (4)
C6—C7—H7	118.0	C14'—C15'—H15'	119.9
N1—C8—C9	104.2 (2)	C16'—C15'—H15'	119.9
N1—C8—H8A	110.9	O3'—C16'—C15'	117.7 (4)
C9—C8—H8A	110.9	O3'—C16'—C11'	124.0 (3)
N1—C8—H8B	110.9	C15'—C16'—C11'	118.2 (3)
			
O5—V1—O1—C1	46.7 (2)	O4—V1—O3—C16	−16.3 (14)
O3—V1—O1—C1	147.7 (2)	V1—O4—C10—C11	−2.3 (18)
O2—V1—O1—C1	−86.3 (3)	O4—C10—C11—C12	178.8 (12)
O3'—V1—O1—C1	149.7 (3)	O4—C10—C11—C16	−6.3 (16)
N1—V1—O1—C1	−44.75 (19)	C16—C11—C12—C13	1.0 (14)
O4'—V1—O1—C1	−127.6 (2)	C10—C11—C12—C13	176.0 (8)
O4—V1—O1—C1	−132.1 (2)	C11—C12—C13—C14	0.4 (12)
O5—V1—O2—C9	−68.2 (2)	C12—C13—C14—C15	−0.2 (12)
O3—V1—O2—C9	−169.3 (2)	C13—C14—C15—C16	−1.3 (13)
O1—V1—O2—C9	64.2 (3)	V1—O3—C16—C15	−166.8 (12)
O3'—V1—O2—C9	−174.3 (3)	V1—O3—C16—C11	14 (2)
N1—V1—O2—C9	22.07 (19)	C14—C15—C16—O3	−177.1 (11)
O4'—V1—O2—C9	106.1 (3)	C14—C15—C16—C11	2.5 (13)
O4—V1—O2—C9	108.7 (2)	C12—C11—C16—O3	177.2 (12)
V1—O1—C1—C2	−147.23 (18)	C10—C11—C16—O3	2.3 (15)
V1—O1—C1—C6	36.1 (3)	C12—C11—C16—C15	−2.4 (13)
O1—C1—C2—C3	−179.7 (2)	C10—C11—C16—C15	−177.2 (9)
C6—C1—C2—C3	−2.9 (4)	O5—V1—O3'—C16'	174.4 (17)
C1—C2—C3—C4	2.1 (4)	O2—V1—O3'—C16'	−81.1 (18)
C2—C3—C4—C5	0.6 (4)	O1—V1—O3'—C16'	76.9 (18)
C3—C4—C5—C6	−2.5 (4)	N1—V1—O3'—C16'	13 (4)
C4—C5—C6—C1	1.7 (4)	O4'—V1—O3'—C16'	−4.7 (17)
C4—C5—C6—C7	176.7 (3)	V1—O4'—C10'—C11'	−8 (2)
O1—C1—C6—C5	177.7 (2)	O4'—C10'—C11'—C12'	−178.7 (16)
C2—C1—C6—C5	1.0 (4)	O4'—C10'—C11'—C16'	6 (2)
O1—C1—C6—C7	2.8 (4)	C16'—C11'—C12'—C13'	−4.1 (17)
C2—C1—C6—C7	−173.9 (2)	C10'—C11'—C12'—C13'	−179.8 (10)
C8—N1—C7—C6	176.5 (2)	C11'—C12'—C13'—C14'	2.4 (15)
V1—N1—C7—C6	−13.5 (4)	C12'—C13'—C14'—C15'	−0.8 (14)
C5—C6—C7—N1	173.2 (2)	C13'—C14'—C15'—C16'	1.0 (16)
C1—C6—C7—N1	−11.9 (4)	V1—O3'—C16'—C15'	−178.2 (15)
C7—N1—C8—C9	141.9 (2)	V1—O3'—C16'—C11'	4 (2)
V1—N1—C8—C9	−29.4 (2)	C14'—C15'—C16'—O3'	179.8 (13)
V1—O2—C9—C8	−45.1 (3)	C14'—C15'—C16'—C11'	−2.6 (17)
N1—C8—C9—O2	44.1 (3)	C12'—C11'—C16'—O3'	−178.5 (15)
O5—V1—O3—C16	157.8 (14)	C10'—C11'—C16'—O3'	−2.9 (19)
O2—V1—O3—C16	−100.1 (14)	C12'—C11'—C16'—C15'	4.1 (17)
O1—V1—O3—C16	58.9 (15)	C10'—C11'—C16'—C15'	179.7 (12)
N1—V1—O3—C16	−37 (3)		

### Hydrogen-bond geometry (Å, º)

Cg is the centroid of the C1–C6 ring.

**Table:**

*D*—H···*A*	*D*—H	H···*A*	*D*···*A*	*D*—H···*A*
C3—H3···O5^i^	0.93	2.56	3.352 (4)	143
C5—H5···O4^ii^	0.93	2.50	3.220 (13)	134
C2—H2···*Cg*^i^	0.93	2.84	3.615 (3)	141

Symmetry codes: (i) −*x*, *y*+1/2, −*z*+1/2; (ii) −*x*+1, *y*−1/2, −*z*+1/2.
