# Supplementary figures and images for: Crystal structure of (2-formyl­phenolato-κ2 O,O′)oxido(2-{[(2-oxidoeth­yl)imino]­meth­yl}phenolato-κ3 O,N,O′)vanadium(V)
Source: Acta Crystallogr E Crystallogr Commun. 2015 Apr 9;71(Pt 5):m104–5. doi: 10.1107/S2056989015006477 (PMC4420105; doi:10.1107/S2056989015006477)

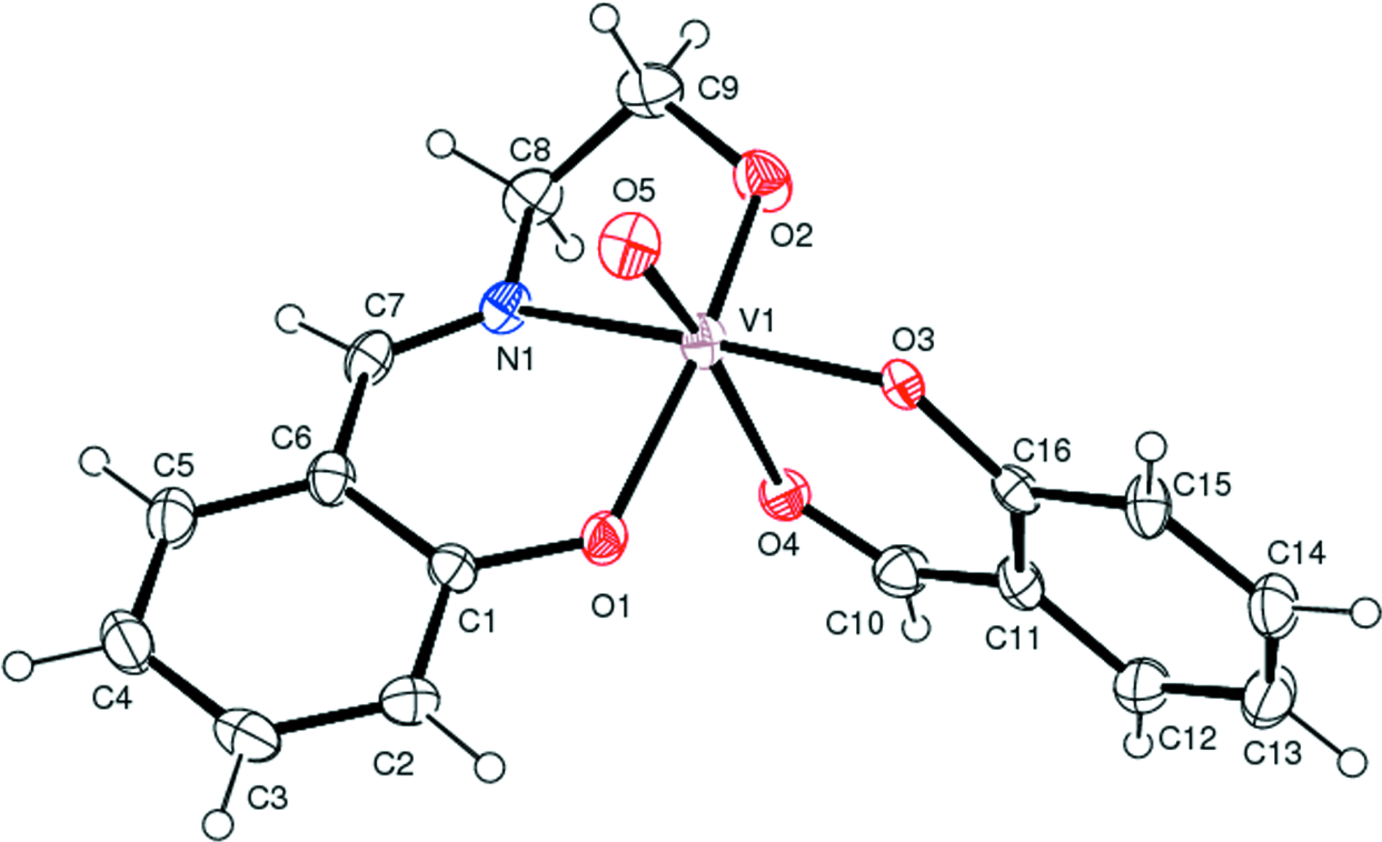

Supplement: Supplementary file 3 [file e-71-0m104-fig1.tif]
